# Transcriptome analysis of halophyte *Nitraria tangutorum* reveals multiple mechanisms to enhance salt resistance

**DOI:** 10.1038/s41598-022-17839-z

**Published:** 2022-08-18

**Authors:** Lirong Wang, Meng Du, Bo Wang, Huirong Duan, Benyin Zhang, Dong Wang, Yi Li, Jiuli Wang

**Affiliations:** 1 Qinghai Provincial Key Laboratory of High-Value Utilization of Characteristic Economic Plants, Qinghai Minzu University, Xining, 810007 China; 2 Institute of Ecology and Environment of Qinghai-Tibet Plateau, Qinghai Minzu University, Xining, 810007 China; 3 College of Forestry, Gansu Agricultural University, Lanzhou, 730000 China; 4 Lanzhou Institute of Husbandry and Pharmaceutical Science, Chinese Academy of Agricultural Sciences, Lanzhou, 730000 China; 5 College of Eco-Environmental Engineering, Qinghai University, Xining, 810016 China; 6 Lanzhou Agriculture and Rural Affairs Bureau in Gansu Province, Lanzhou, 730030 China

**Keywords:** Molecular biology, Physiology, Plant sciences

## Abstract

As a typical halophyte, *Nitraria tangutorum* Bobr. has attracted the interest of many researchers with the excellent salt tolerance. Elucidation of the mechanism of *N. tangutorum* salinity tolerance will facilitate the genetic improvement of productive plants faced with salinity. To reveal the molecular response to gradually accumulated salt stress in *N. tangutorum*, RNA-sequencing and analysis of gradually accumulated NaCl treated samples and control samples were performed, and a total of 1419 differentially expressed genes were identified, including 949 down-regulated genes and 470 up-regulated genes. Detailed analysis uncovered that the catabolism of organic compounds mainly based on oxidative phosphorylation genes was up-regulated. Additionally, various antioxidant genes, especially anthocyanin-related genes, were found to help *N. tangutorum* remove reactive oxygen species. Moreover, the Mitogen activated protein kinase signaling pathway and other signaling pathways co-regulated various salt tolerance activities. Additionally, intracellular ion homeostasis was maintained via regulation of osmotic regulator-related genes, cutin-related genes, and cell elongation-related genes to retain cellular water and reduce ion concentration. In particularly, simultaneous up-regulation in cytoskeleton-related genes, cell wall-related genes, and auxin-related genes, provided evidence of important role of cell expansion in plant salt tolerance. In conclusion, complex regulatory mechanisms modulated by multiple genes might contribute to the salt tolerance by *N. tangutorum*.

## Introduction

Soil salinization, which is widespread worldwide because of climate change and excessive irrigation, threatens plant growth, crop production, and ecological stability^1,2^. Accordingly, determining how to harness saline land is a global problem. Halophytes, which are naturally occurring in saline habitats, have developed multiple adaptive mechanisms that allow them to survive and grow well in saline environments^3^. Elucidation of the mechanisms responsible for halophyte salinity tolerance will facilitate genetic improvement of productive plants that are tolerant of salinity while also helping to harness saline soil^3^.

*Nitraria tangutorum* Bobr., which belongs to the Zygophyllaceae family, is a halophytic shrub widely distributed in the saline soil of arid and semi-arid areas, lakesides, and coastal areas in China^4–6^. As one of the most salt-tolerant shrubs, two-month-old *N. tangutorum* seedlings did not induce any evident phenotypic differences in leaves when they were treated with 500 mM NaCl for 3 days^6^; however, many glycophytes showed growth inhibition following exposure to NaCl at above 100 mM^7,8^. Due to its superior tolerance of high salinity, *N. tangutorum* is an excellent material for studying mechanisms of plant salt tolerance. To date, the mechanisms responsible for *N. tangutorum* salt tolerance have been investigated at the morphological, anatomic structural, physiological, and molecular levels^4–6,9^. Notably, understanding molecular mechanisms is important for development of effective genetic engineering strategies to enhance tolerance against salt stress in crops, pastures, and forests, which will improve the biological yield of saline land and prevent desertification caused by salinification.

Response against salinity stress involves a complex multitude of activities operating in close coordination^3^. Halophytes have evolved specific strategies for salt tolerance, including the adjustment of energy metabolism, activating antioxidant systems to reduce stress damage, and balancing ion homeostasis through various methods^3,10^. These activities involve thousands of genes. Identifying salt-tolerant genes and analyzing their interaction will help elucidate the molecular basis behind specific biological events involved in halophyte salt tolerance.

Comparative transcriptome analysis is an effective tool for identifying candidate genes associated with various environmental stresses based on recognition of potential functions of differentially expressed genes (DEGs), as well as the regulatory and metabolic pathways involved in tolerance^10^. Although an earlier study of salt tolerance mechanisms in *N. tangutorum* was conducted using comparative proteomics^6^, comparative transcriptome analysis will provide more information. Moreover, because of the wide range of salt-tolerance, halophytes show multiple methods of dealing with different concentrations of salt stress^3,11^. In addition, different treatments (e.g., gradually increased salinity or salt shock) also lead to different coping patterns and physiological characteristics in halophytes^12^. Therefore, in the present study, *N. tangutorun* plants were exposed to up to 350 mM NaCl gradually (salt acclimation). A gene expression map of *N. tangutorun* responding to gradual step acclimation salt treatments was then created. The results presented herein will contribute to future practices of genetic improvement of plants and the ecological conservation of salinized areas.

## Materials and methods

### Plant material culture and treatment

Seeds of *N. tangutorum* were collected from Nomuhong Farm Golmud, Qinghai Province, China, (N36° 57′ 8.72″, E102° 28′ 54.94″), and stored at 4 °C until use. The collection of plant material was requisite permission from the farm administrator and complied with relevant regulations. The voucher specimen was identified by an expert on plant taxonomy from Qinghai Society of Botany (Jiuli Wang) and deposited in Herbarium of the College of Ecological Environment and Resources, Qinghai Minzu University (Wang-20200729-005). For analysis, seeds were surface-sterilized with 75% alcohol for 30 s, then washed with sterile water three times, after which they were germinated in individual plastic pots filled with washed river sand that had been sterilized at 121.3 °C and 103.4 kPa pressure for 15 min^6^. After initial irrigation with 1/2 Hoagland nutrient solution, pots were placed under natural light at room temperature and irrigated every three days with deionized water from the bottom of the planter tray. After 30 days, seedlings were transferred to a plant incubator with a temperature of 26/23 °C (day/night), relative humidity of 60–70%, and daily photoperiod of 16/8 h (light/dark; flux density 400–600 μmol m^−2^ s^−1^).

After 14 days, uniform seedlings were divided into a control and treated group. Control plants were irrigated every day with deionized water as described above, while other plants were subjected to salt treatments. To avoid the risk of plasmolysis due to the osmotic shock and closely reflects natural incidences of salinity, the "gradual step acclimation” method was used in our experiment^12,13^. Salt application started with 50 mM on day 1 and was then increased 50 mM per day until reaching 350 mM on day 7. All shoots were sampled 2 h after the final irrigation, at which time they were flash-frozen in liquid nitrogen and stored at − 80 °C until analysis. Plants used for quantitative reverse transcription polymerase chain reaction (qRT-PCR) validation were cultured and treated as described above.

### Library construction, sequencing, and de novo assembly

Total RNA was isolated using an RNAprep Pure Plant kit (Tiangen, Beijing, China) with a DNase treatment step. After RNA quality assessment (RIN value > 7), six cDNA libraries (control sample-1: CK-1, control sample-2: CK-2, control sample-3: CK-3, salt treated sample-1: S1, salt treated sample-2: S2, salt treated sample-3: S3) from three seedlings treated with NaCl and three control plants were constructed and validated on an Agilent Technologies 2100 Bio-analyzer, then sequenced on the Illumina HiSeq™ 2000 platform and 125-bp paired-end reads were generated.

Following sequencing quality assessment, poor quality sequences were removed, and adapter sequences were trimmed from the raw sequence data using Cutadapt (v1.8.1)^14^. The clean data for each sample were then assembled into transcripts using the Trinity software with the default parameters^15^. Subsequently, all assembled transcripts were clustered by Cd-hit (v4.6) using the default parameters to filter redundancies^16^. Additionally, the longest transcript in each gene cluster defined by Trinity was selected as a “unigene”^17^. Finally, unigenes with fragments per kilobase of exon model per million mapped fragments (FPKM) values ≥ 2 in at least one sample were screened for downstream analysis.

### Functional annotation

The amino acid sequences of selected unigenes were predicted using Transdecoder (v.2.0.1) with the default parameters^18^. Next, the amino acid sequences were subjected to BLASTX (E-value ≤ 1e^−5^) searches of the following protein databases: Non-redundant (Nr, https://ftp.ncbi.nlm.nih.gov/blast/db/FASTA/)^10^, EggNOG (http://eggnog5.embl.de/#/app/emapper)^19^, InterProScan (IPR, https://www.ebi.ac.uk/interpro/search/sequence/), and Kyoto Encyclopedia of Genes and Genomes (KEGG, https://www.kegg.jp/)^17^.

### Identification and enrichment analysis of DEGs

Differentially expressed genes across samples were identified according to their expression levels with the thresholds of |log_2_ (treated group/control group)|≥ 1 and p-value < 0.05^20^. Next, Gene Ontology (GO) and KEGG enrichment analyses of *N. tangutorum* DEGs were conducted using the GO and KEGG databases to determine the GO functional classification and KEGG pathway annotation, after which a hypergeometric test was used to identify significantly enriched GO terms and KEGG pathways^21^. If the p-value was < 0.05 and the Enrichment Score was < 10, the GO terms were regarded as significantly enriched, KEGG pathways were filtered and enriched by the DEGs with the cutoff *p* < 0.05^20^. Itak (http://bioinfo.bti.cornell.edu/tool/itak) with an E-value cutoff of 10^−5^ was employed to predict transcription factors (TFs)^22^.

### qRT-PCR

To confirm the gene expression inferred from RNA-seq, a total of 10 DEGs were randomly selected for qRT-PCR analyses. The *elongation factor 1-alpha* (*EF1-α*) gene of *N. tangutorun* was used as an internal control^23^. Gene-specific primers were designed using the online software OligoArchitect (http://www.oligoarchitect.com/LoginServlet) (Supplementary Table S1) and synthesized by Sangon Biotech Co., Ltd. (Shanghai, China). Total RNA was extracted from the six samples (in triplicate) as described above, after which cDNA was synthesized using an Evo M-MLV RT Premix for qPCR Kit (Accurate Biology Co., Ltd., Shanghai, China). qRT-PCR was then performed using an ABI ViiA 7 Real-Time PCR System (Applied Biosystems, Carlsbad, USA) and SYBRGreen Mastermix (Qiagen, California, USA). The relative expression levels of genes were calculated using the 2^−ΔΔCt^ method^10,20^.

### Statistical analysis

RNA-Seq and qRT-PCR analysis were performed on the samples described above with three biological replicates for each. All qRT-PCR data were expressed as means ± SEs (*n* = 3). Statistical analyses were performed using SPSS 21.0 software, and One-way ANOVA method was used to conduct significant difference analysis on the relative expression levels of the validated genes (*p* < 0.05).

## Results

### De novo assembly and quantitative assessment of Illumina sequences

To obtain complete gene expression information for *N. tangutorun*, six RNA-seq libraries of shoots collected from *N. tangutorun* plants treated with gradual step acclimation salt and control plants were generated. Overall, 277.8 million clean reads of 125 bp with an average Q30 of 91.64% and an average GC percentage of 45% were generated. Sequencing quality assessment showed that the GC and AT contents were equal in each sequencing cycle, showing that the entire process was stable and unchanged, and so presented a horizontal line (Supplementary Fig. S1), which was expected for a randomly fragmented transcriptome. These findings suggested that the sequencing output and quality were adequate for further analysis (information about mapping and sample expression quality are shown in Supplementary Table S2 and Supplementary Figs. S2–5, respectively).

Subsequently, clean reads were assembled, and 101,596 transcripts (deposited in the China National GeneBank Sequence Archive (CNSA, https://db.cngb.org/cnsa/) of China National GeneBank DataBase (CNGBdb) with accession number CNP0001137.) with an average length of 846 bp, an average GC percentage of 39.91%, and an N50 length of 1544 bp were generated. Statistical summary of sequencing and assembly results were displayed in Table 1. After clustering, 83,642 unigenes were identified. The length distributions of transcripts and distribution of unigene numbers for transcripts are shown in Supplementary Figs. S6–7.

**Table 1:** Statistical summary of sequencing and assembly results.

Items	CK-1	CK-2	CK-3	S-1	S-2	S-3
Clean raw reads	46,746,490	41,319,628	45,872,422	50,644,394	49,170,238	44,037,538
Q20 value (%)	95.38	95.37	96.13	96.28	95.34	95.22
Q30 value (%)	91.21	91.27	92.47	92.73	91.14	91
GC content (%)	45	45	45	45	45	45
LowQ (< 5) (%)	0.81	0.83	0.625	0.57	0.885	0.88
Total assembled bases	86,017,411					
Average contig	846					
Median contig	448					
Total transcripts	101,596					
Total unigenes	83,655					
GC content fo total Transcripts (%)	39.91					
N50 of total transcripts (bp)	1544					

**Figure 1:**
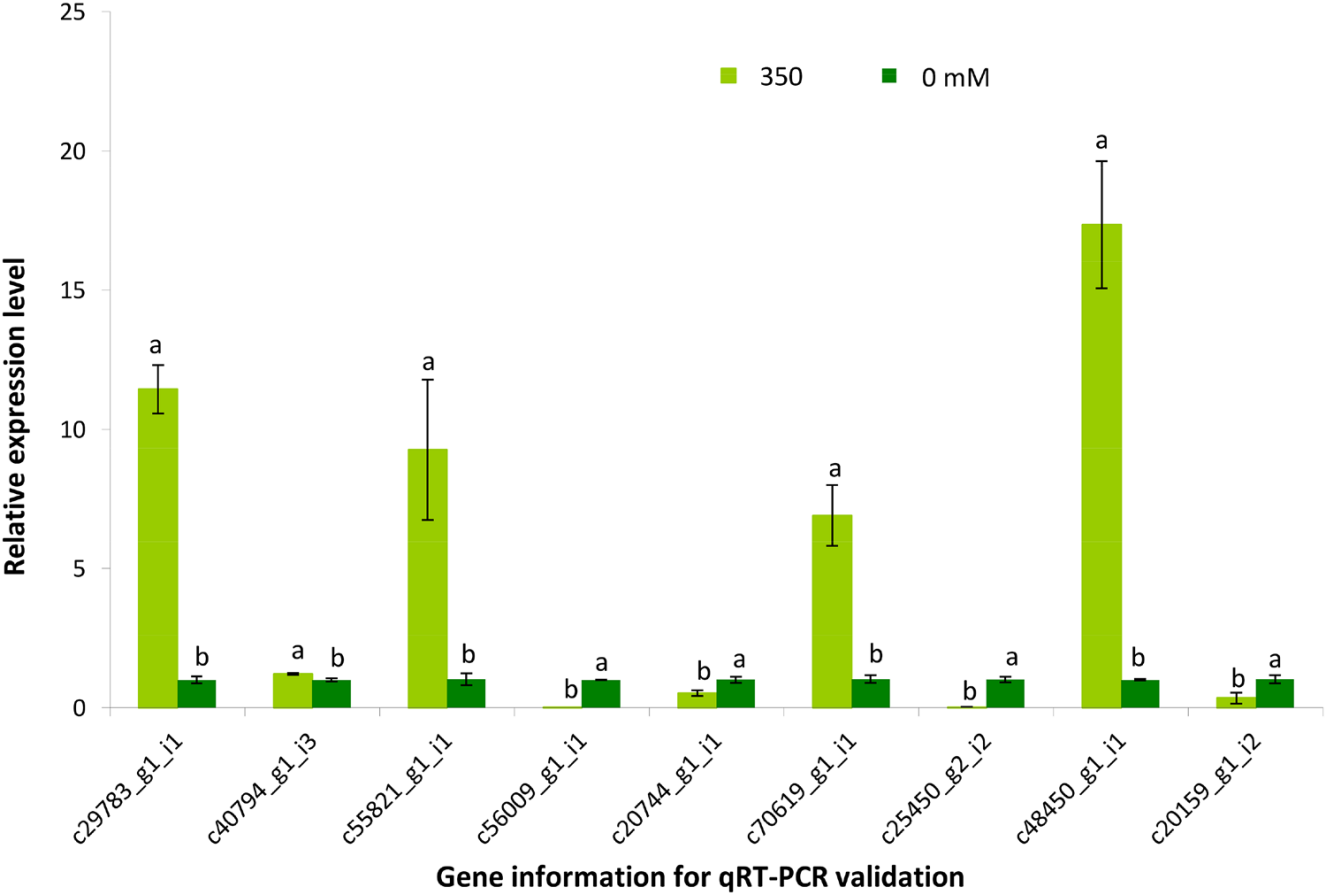
Validation of differentially expressed genes (DEGs) obtained from comparison of 350 mM NaCl treated plants shoots and control plants shoots using qRT-PCR. Each panel shows the qRT-PCR results for nine amplified genes. The gene information for qRT-PCR validation is listed on the X-axis and the mean fold changes represented by the 2^−ΔΔCt^ method relative to 0 mM treated samples are shown on the Y-axis. Error bars depict the standard error of the mean for three biological replicates. Significant differences between 350 mM NaCl treated plants and control of each gene (*p* < 0.05) are denoted with lowercase “a” and “b”.

To validate the assembly quality of *N. tangutorum* unigenes, ten DEGs were randomly selected for qRT-PCR, and nine of these were successfully amplified (Fig. 1). In addition, the tendency of transcriptional changes of all tested genes was consistent between the two methods, suggesting that the assembled transcripts were accurate (the correlation between both methods are shown by the scatter plot in Supplementary Fig. S8). Among the nine genes, four genes were down-regulated including a TF (c20159_g1_i2, auxin response factor, ARF), a protein mediating the ubiquitination involved in cell cycle progression (c25450_g2_i2, S-phase kinase-associated protein 1, SKP1), a DNA/RNA polymerase (c20744_g1_i1), and a Cytochrome (c56009_g1_i1); five genes were up-regulated including a DNA/RNA polymerase (c29783_g1_i1), a novel signalling motif (c40794_g1_i3, NB-ARC domain), a chlorophyll degradation related enzyme (c55821_g1_i1, chlorophyllase, Clh), a molecular motor (c70619_g1_i1, myosin), and a cytoskeleton related gene (c48450_g1_i1, actin).

### Functional annotation

Functions of the 48,274 unigenes with FPKM values ≥ 2 were annotated by blasting against the public databases with the cutoff of E-value < 1e^−5^. The most matches (20,962, 43.4%) were obtained upon alignment with the Nr database (Supplementary Table S3). The remaining unigenes could not be annotated with known genes, most likely because of an absence of genome information or technical limitations, such as inadequate read length and sequencing depth. Based on Nr annotations, 96.63% of the sequences were mapped to the known genes in plants with E-values of < 1e^−20^ (Supplementary Fig. S9a), and 63.43% of the annotated unigenes matched deposited sequences with a similarity of > 70% (Supplementary Fig. S9b). Analysis of the species distribution revealed that the top three matches for the *N. tangutorum* unigenes were *Citrus sinensis* (37.82%), *Citrus clementina* (21.60%), and *Theobroma cacao* (7.63%) (Supplementary Fig. S9c).

**Figure 2:**
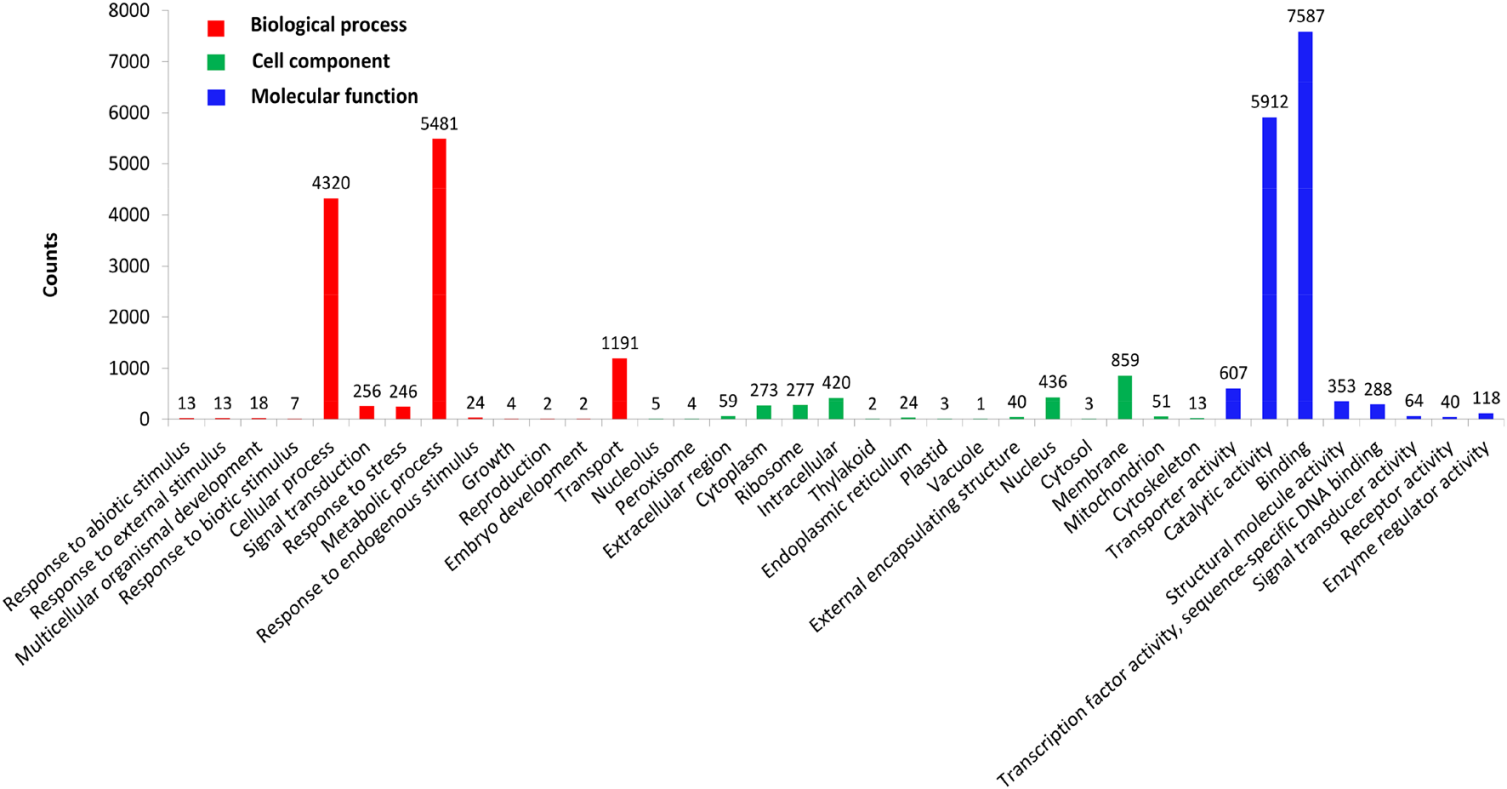
Unigene GO classifications. The Y-axis represents the number of a specific term of genes in each main category (Biological Process, Molecular Function, and Cellular Components) and the X-axis represents the 37 terms.

Searching against the GO, EggNOG, and KEGG databases provided functional classification or pathway information for the unigenes. Overall, 12,947 unigenes were assigned at least one GO term to a total of 2970 GO annotations. The annotations fell into three main categories: Biological Processes (11,577), Cellular Components (2470), and Molecular Function (14,969) (Fig. 2). Other annotation results are displayed in Supplementary Tables S3–S8, and annotation summary is shown in Supplementary Table S9.

### DEGs functional annotation and classification

To identify DEGs under salt treatment, normalized FPKM (Supplementary Table S10) was used to quantify the transcript levels, after which differences in gene expression were evaluated by salt stress libraries and comparing control libraries. In total, 1419 DEGs were identified using the thresholds described above. Among these, 949 DEGs were up-regulated and 470 DEGs were down-regulated (Supplementary Table S11).

**Figure 3:**
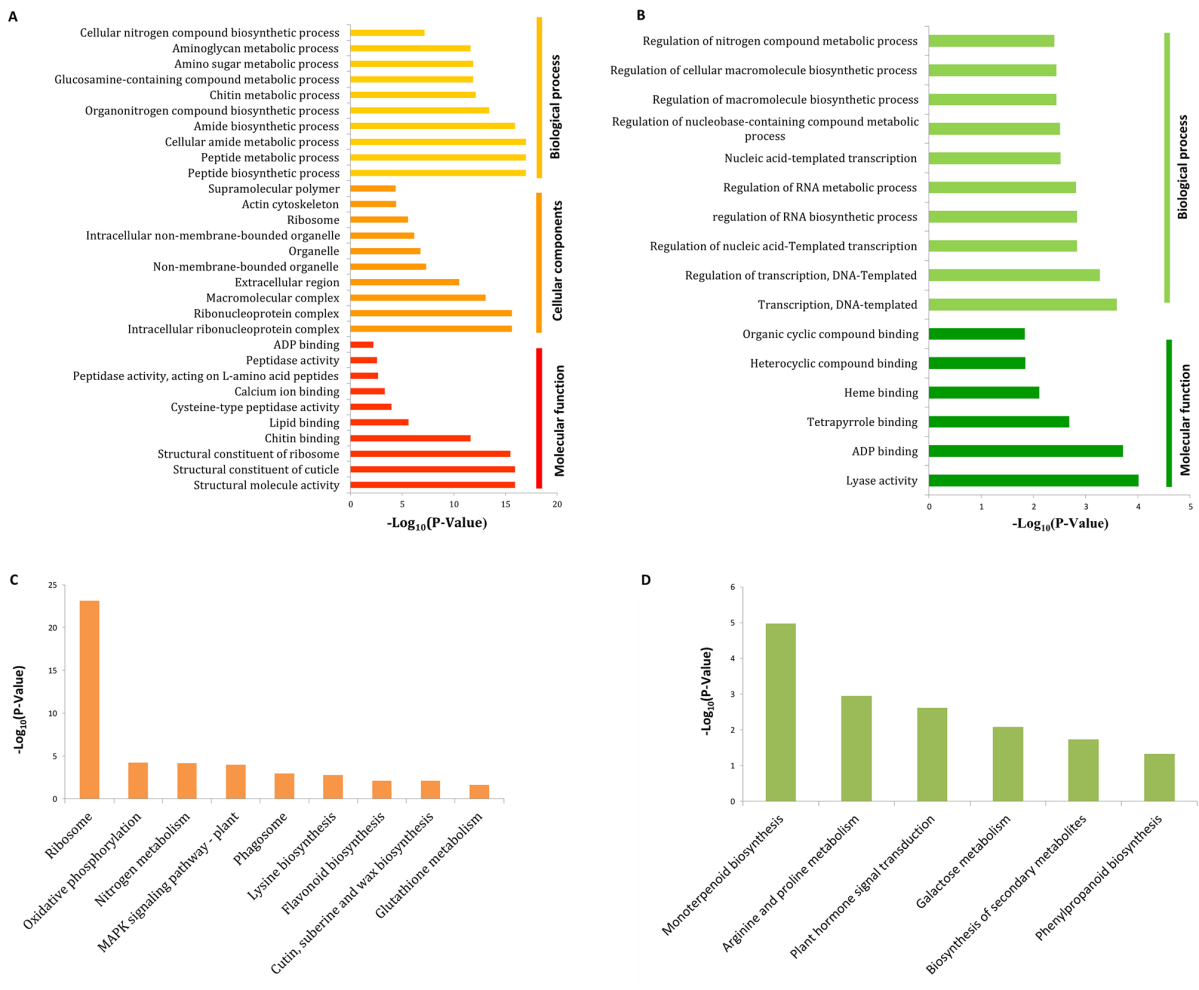
The main results of differential gene enrichment analysis. The TOP 10 significantly enriched GO terms in each main categories for (**A**) up and (**B**) down-regulated genes (or be short of 10 terms). Significantly enriched KEGG pathways for (**C**) up and (**D**) down-regulated genes.

Enrichment analysis can screen out more information such as important physiological processes than bare functional annotation, facilitating analysis of the relatively comprehensive response mechanism to plants under stress^17^. Therefore, we conducted enrichment analysis using GO and KEGG for up-regulated genes and down-regulated genes. GO enrichment analysis showed that a total of 136 terms were significantly enriched, including 108 up-regulated (Supplementary Table S12) and 31 down-regulated genes (Supplementary Table S13). The up-regulated genes were assigned to the categories of Biological Process, Molecular Function and Cellular Components (Fig. 3a), while the down-regulated genes were only assigned to the first two categories (Fig. 3b). Overall, 83 KEGG pathways were identified. Enrichment analysis of up-regulated genes revealed 75 KEGG pathways (Supplementary Table S14), including nine significantly enriched pathways (Fig. 3c); namely, “ribosome,” “oxidative phosphorylation,” “nitrogen metabolism,” “mitogen-activated protein kinase (MAPK) signaling pathway—plant,” “phagosome,” “lysine biosynthesis,” “flavonoid biosynthesis,” “cutin,” and “suberine and wax biosynthesis.” For down-regulated genes, 39 KEGG pathways were identified (Supplementary Table S15), with the following six being significantly enriched (Fig. 3d): “monoterpenoid biosynthesis,” “arginine and proline metabolism,” “plant hormone signal transduction,” “galactose metabolism,” “biosynthesis of secondary metabolites,” and “phenylpropanoid biosynthesis.”

## Discussion

### Energy metabolism

**Figure 4:**
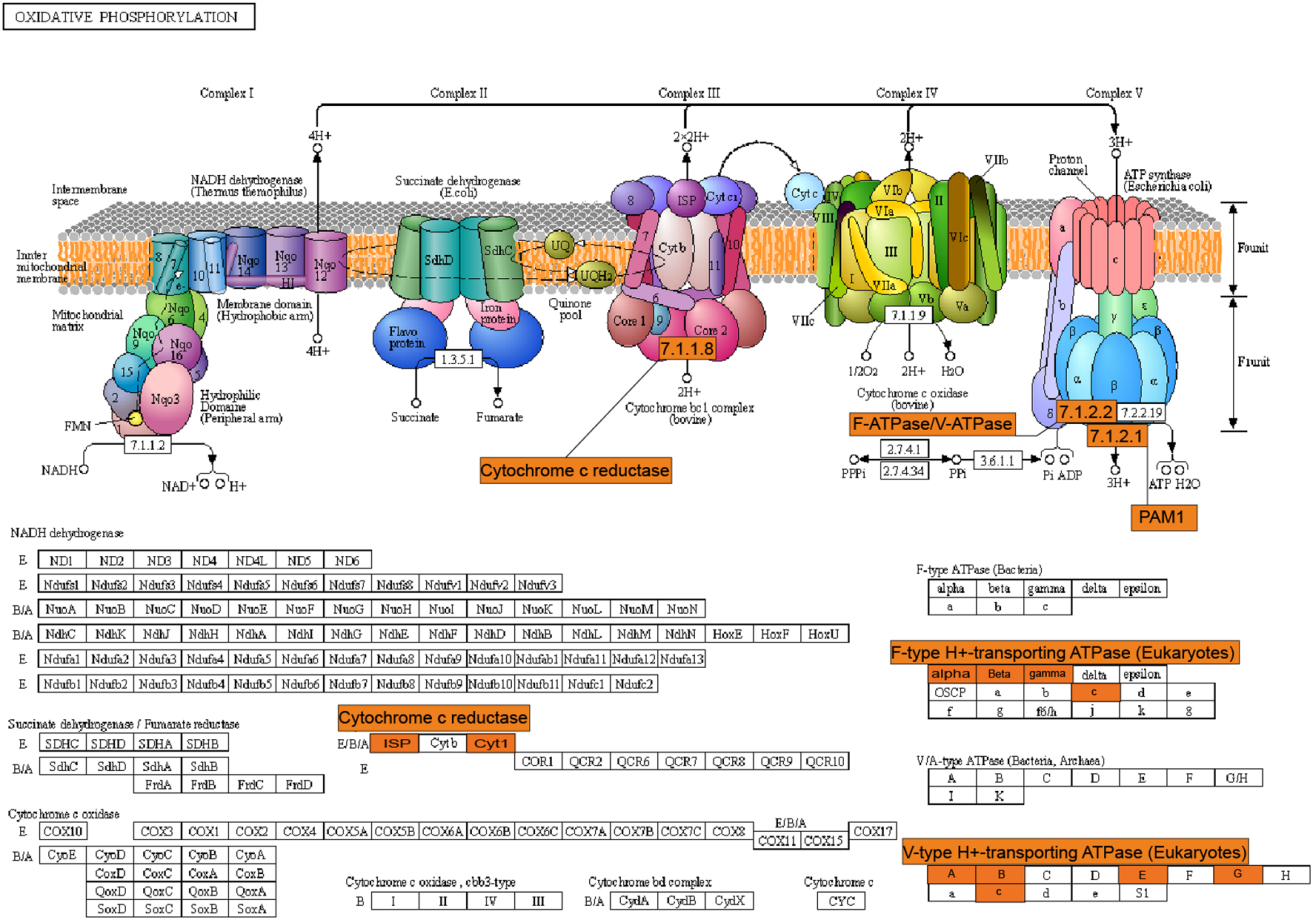
Overview of salinity stress-responsive genes involved in OXPHOS in *N. tangutorum* plants^26^.

Energy metabolism is one of the most basic characteristics of life, and increasing transcriptome data indicate a relatively robust metabolism is needed to cope with environmental pressure^21^. In this study, most of the genes associated with organic compound catabolism (cellular respiration) pathways were found to be up-regulated. In the “glycolysis” pathway, genes encoding enolase (ENO), fructose-bisphosphate aldolase (ALDO), and genes in the anaerobic respiratory branching pathway, including *aldehyde dehydrogenase* (*ALDH*) and *alcohol dehydrogenase* (*ADH*), were up-regulated by about 7-fold. In addition, genes encoding malate dehydrogenase (MDH1) and citrate synthase (CS) belonging to the “citric acid cycle (TCA)” pathway were found to be up-regulated. However, the gene encoding lsocitrate lyase (ICL) in this pathway was down-regulated. KEGG enrichment analysis indicated that “oxidative phosphorylation (OXPHOS)” was the most enriched pathway for up-regulated genes. In the respiratory transport chain of OXPHOS, two genes encoding the ubiquinol-cytochrome c reductase cytochrome b/c1 subunit (Cyt1) and the ubiquinol-cytochromec reductase iron-sulfur subunit (ISP), which make up the second protein complex, were up-regulated. Moreover, eight *F-type H*^+^*-transporting ATPase* (*F-ATPase*) homologous genes, five *V-type H*^+^*-transporting ATPase* (*V-ATPase*) homologous genes and one gene encoding the H^+^-transporting ATPase (PAM1/2) on the last protein complex were highly up-regulated. F-ATPase catalyzes synthesis of ATP during OXPHOS utilizing the energy of an electrochemical H^+^ gradient (A ~ H^+^) generated by electron transport^24^. The related enzyme V-ATPase couples ATP hydrolysis with acidification of a variety of vesicles^24^, which is essential for establishing an electrochemical H^+^-gradient across tonoplasts to energize tonoplasts for efficient ion uptake into vacuoles via the tonoplast Na^+^/H^+^ antiporter (NHX)^10^. Similar to our results, many transcriptome data have shown that *F-ATPase* and *V-ATPase* were up-regulated under salt stress, and increasing activity of proteins they encode has been found to contribute to improvements in salt tolerance in plants^10,21,25^. Although there were a small number of down-regulated genes found in cellular respiration pathways, overall catabolic activity was enhanced, and OXPHOS in particular seemed to play a more important role (Fig. 4).

In addition to enhancing of cellular respiration, genes in other pathways associated with cellular respiration or organic metabolism exhibited differential expression. For example, *Transketolase* (*TktA/B*) belonging to the “pentose phosphate” pathway and two *beta-glucosidase* (*β-glu*) homologous genes belonging to the “starch and sucrose metabolism” pathway were up-regulated. Of course, not all genes involved in organic metabolism were up-regulated. For example, one of *pectate lyase* (*PLY*) genes and *acetyl-CoA carboxylase* (*ACC*) genes, which belong to the “pentose and glucuronate interconversions” pathway and “pyruvate metabolism” pathway, respectively, were down-regulated. This down-regulation may be related to their other roles in stress response; specifically, *PLY* might be related to cell wall activities and *ACC* might be related to fatty acid synthesis^27,28^. In addition to metabolism of organic compounds containing carbon, some genes involved in the decomposition of organic compounds containing nitrogen and sulfur were also up-regulated. Specifically, the gene encoding *cysteine synthase* (*cysK*), which is downstream of the “sulfur metabolic” pathways, was up-regulated, as were seven *Nitrite transporter* (*Nrt*) and two amino acid synthesis genes [*Glutaminesynthetase* (*GLUL*) and *Glutamate dehydrogenase* (*GLUD*)], which are distributed in the upstream and downstream portions of the “nitrogen metabolism” pathway, respectively.

The photosynthetic system is an important part of the total energy metabolism of plants. In this study, four genes associated with photosynthesis were found to be differentially expressed. Specifically, the gene encoding Photosystem II chlorophyll binding (PsbB) was slightly down-regulated, while the gene encoding Clh, which forms part of the chlorophyll degradation pathway^29^ was dramatically up-regulated. Moreover, the gene encoding carbonic anhydrase (CA), which is well known to reversibly convert CO_2_ into HCO_3_^–^ in photosynthesis-related activities and help improve the efficiency of photosynthesis, was down-regulated^30^. Accordingly, changes in these genes would inhibit photosynthesis. However, the genes encoding Far-red impaired response1 (FAR1) were up-regulated, which regulate chlorophyll biosynthesis and would benefit photosynthesis. Moreover, the observed changes in these genes indicated that the inhibition of photosynthesis is not because of component damage, but rather the result of positive regulation. Taken together, genes in the photosynthetic system provide a conflicting picture, with the photosynthetic system attempting to accumulate energy substances while also being regulated to decrease photosynthesis when subjected to saline conditions in this study.

The data of ribosomal gene laterally proved energy-consuming increased. KEGG enrichment analysis showed that the “ribosome” pathway was significantly enriched, 54 genes including a proportion of genes encoding small subunit protein genes and a proportion of genes encoding large subunit protein genes were significantly up-regulated (Supplementary Fig. S10). It is similar to the results of Fu et al., in their study on *Shewanella Algae*, ribosomal genes were highly expressed in the early stage of salt stress^31^. It is well known that ribosome synthesis requires ATPase, GTPase and so on, which is a process of high energy consumption^31–33^ Therefore, a large number of ribosome gene syntheses can also prove from the side that energy consumption of *N. tangutorum* is enhanced in the early response to salt stress.

In this study, energy metabolism data indicated that anabolism was weakened, while catabolism mainly based on OXPHOS was strengthened. This form of catabolism is very important to supplying ATP used for ion transport, signal transduction, ribosomal biosynthesis, and other salt tolerant activities. Information about DEGs involved in energy metabolism is given in Supplementary Table S16.

### Antioxidant activity

**Figure 5:**
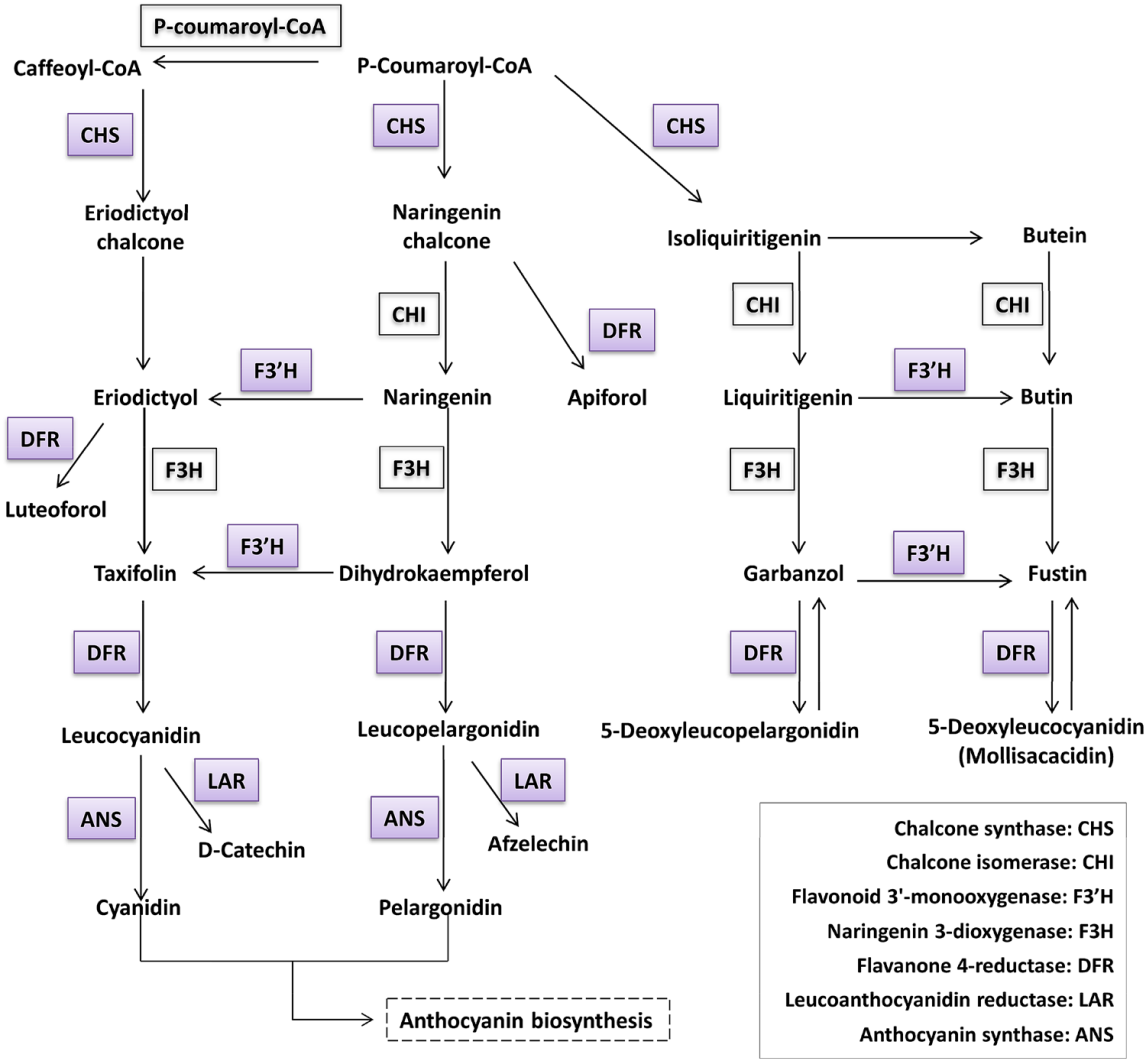
A putative flavonoid synthesis pathway, which was generated from the KEGG database based on the log_2_ (fold change) values of identified differentially expressed genes. Colored boxes represent up-regulated genes. The symbols in boxes indicate the gene codes.

Salt stress can increase the production of reactive oxide species (ROS), causing oxidative damage to cellular structures^1^. Antioxidants such as flavonoids, carotenoids, glutathione (GSH), ascorbic acid, and antioxidant enzymes play important roles in protecting plants from oxidative damage caused by salt stress^10^. In this study, some non-enzymatic, antioxidant-related genes were up-regulated. KEGG enrichment analysis showed that the “flavonoid synthesis” pathway was significantly enriched. A series of genes in this pathway were up-regulated, including the upstream gene *chalcone synthase* (*CHS*), midstream gene *flavonoid 3'-monooxygenase* (*F3’H*) as well as the downstream genes *flavanone 4-reductase* (*DFR*) and *leucoanthocyanidin reductase* (*LAR*). Among them, DFR can activate the biosynthetic precursors of anthocyanin^30^. Moreover, an anthocyanin synthesis gene, *Oxoglutarate*/*iron-dependent dioxygenase* (*2-GO*), was annotated by GO and highly up-regulated, which catalyzes the penultimate step in the biosynthesis of the anthocyanin class of flavonoids, from the colorless leucoanthocyanidins to the colored anthocyanidins^35^ (Fig. 5). Anthocyanins are natural colorants belonging to the flavonoid family that had been shown to possess potent antioxidant properties^36^. Up-regulation of anthocyanin-related genes indicated that anthocyanins play an important role in ROS scavenging in *N. tangutorum* under salt stress.

In addition to the genes related to flavonoids, other antioxidant genes varied. A carotenoid-related gene, *9-cis-epoxycarotenoid dioxygenase* (*NCED*), which converts carotenoid into other substances and a gene associated with GSH hydrolase were both found to be down-regulated, which led to accumulation of carotenoid and GSH, respectively. Six *Glutathione S-transferase* (*GST*) genes were detected in transcriptome, all of which were up-regulated. GST can prevent the degradation of organic hydroperoxides to cytotoxic aldehyde derivatives by catalyzing coupling reactions between processed products and GSH^10^. Genes encoding thioredoxins (Trxs) were up-regulated by 6- to 8-fold. Trxs are known to be involved in the regulation of the cell REDOX environment and the repair of protein and DNA^37^. However, *glutaredoxinA*s (*GrxA*s) belonging to the *Trx* family showed a decrease^38^. This indicated that *Trx* family genes play different roles in *N. tangutorum* under salt stress. Haem peroxidases (HRPs), a kind of enzyme antioxidant that acts as a receptor for hydrogen peroxide^39,40^, were also found in our transcriptome. One of the genes associated with this enzyme was up-regulated by 1.3-fold, while the other two were down-regulated by about 1.6-fold. The HRP family also showed functional differentiation in this study. Overall, there were more non-enzymatic, antioxidant-related DEGs than enzymatic, antioxidant-related DEGs found in our transcriptome, and they seemed to play an important role in salt tolerance of *N. tangutorum* under the investigated conditions, especially anthocyanins. Information about DEGs involved in antioxidant activity is given in Supplementary Table S17.

### Ion homeostasis regulation

**Figure 6:**
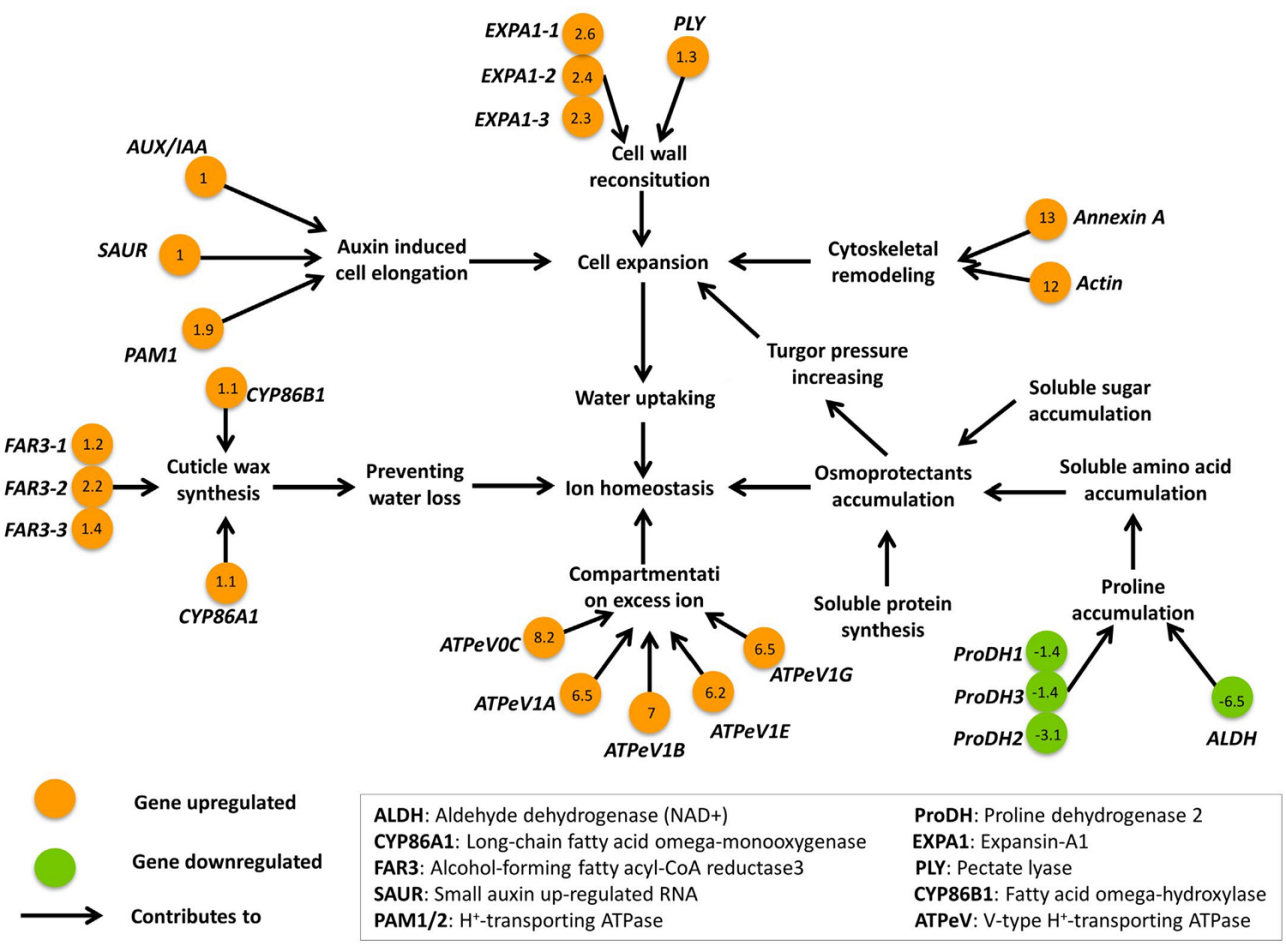
Global view of the identified differentially expressed genes (DEGs) involved in ion homeostasis regulation. Orange and green colors represent up and down-regulated DEGs, respectively. The numbers in the circles represent the fold change.

The adjustment of ion homeostasis under salt stress conditions is an important strategy to cope with salinity stress^41^. Salinity will cause osmotic stress and limitation of water absorption, then lead to disruption of ion homeostasis and ion toxicity. Several approaches have evolved to balance ion homeostasis in halophytes, including Na^+^/H^+^ antiporters and transporters (K^+^ and Cl^−^) employed to compartmentalize excess ion into vacuoles and/or in less sensitive tissues; reduction of water loss through closing stomas or increasing cuticles; and osmotic potential changes via synthesis of osmoregulation components such as proline, glycine betaine, soluble sugar, and amino acids. Moreover, aquaporins and ion channel proteins in plant roots are responsible for water absorption and controlling the entry of ions, respectively^41^. A global view of the identified DEGs involved in ion homeostasis regulation is shown in Fig. 6.

Transporting ion into vacuoles under salt stress is an important strategy for plants that balance ion homeostasis. NHXs and ATPase are reported to be the two main types of protein to accomplish this strategy^42^. As mentioned above, V-ATPase provides energy for NHXs on tonoplasts, and the genes encoding this type of enzyme were found to be highly up-regulated in the present study. However, no significant up-regulation of *NHXs* was found in our study. Moreover, our expression data showed that *NHXs* had a relatively high expression level in control plants (FPKM value: 25–28), and their expression increased slightly (FPKM value: 29–35) but not statistically significantly under salt stress. Similar results have been reported in other studies. In *Arabidopsis*, vacuolar NHXs were overexpressed, but no increase in *AtNHX1* transcript levels was detected in response to NaCl stress at 50–250 mM^43^. In addition, no significant up-regulation was observed in the transcriptome of a halophyte (*Halogeton*) treated with low levels of salt (200 mM)^10^. Wang’s explanation for the above results is that increase in vacuolar *NHX* requires a higher concentration of NaCl in the halophyte *Halogeton glomeratus*^10^. On that basis, we found in both transgenic *Arabidopsis thaliana* and in our study, *NHXs* were naturally expressed at relatively high expression levels; therefore, a further assumption is that low concentrations of salt could not stimulate the up-regulation of *NHXs* in plants that are naturally expressed at relatively high expression levels. This viewpoint is in agreement with, and more in-depth than, Wang’s previous study in *Halogeton.*

Many studies have shown that halophytes have thick cuticles that are involved in their adaptation to highly saline environments^44–46^. In this study, KEGG enrichment analysis revealed that the cuticle wax synthesis pathway was significantly enriched, and that five upstream genes in the pathway were up-regulated (one *Long-chain fatty acid omega-monooxygenase* (*CYP86A1*) gene, one *Fatty acid omega-hydroxylase* (*CYP86B1*) gene, and three *Alcohol-forming fatty acyl-CoA reductase3* (*FAR3*) genes). In fact, *N. tangutorum* has been shown to accumulate cuticle wax during salt stress^47^. Although the comprehensive molecular mechanism of the adaptive phenotype has still not been elucidated, our data provide molecular evidence of this phenotype. The wax accumulation is likely helpful for preventing water loss, osmotic regulation is also an important way through which many plants prevent water loss under salt stress^41^. Osmoregulators such as proline, betaine, soluble sugars, and soluble proteins were regulated to accumulate, then increase water-holding capacity and cell turgor pressure^41^. Three *proline dehydrogenase* (*ProDH*) genes were found to be down-regulated, which promoted the accumulation of proline. In addition, enrichment analysis revealed that pathways related to carbon and nitrogen metabolism, including “glycolysis,” “TCA,” “OXPHOS,” “lysine biosynthesis,” and “glutathione metabolism,” were significantly enriched. Carbon and nitrogen metabolism produces many derivatives of sugar, amino acids, and proteins, most of which are osmotic regulators.

Cell enlarging activities appear to be related to salt resistance. Expansin (EXP) is a cell wall relaxation protein that is closely related to plant growth^48^. Recently, *EXPAs* were demonstrated to be up-regulated by salt stresses, and improvement of salt tolerance has been shown in response to overexpression of a wheat *EXPA* and other plants *EXPA*s^48,49^. In this study, three *EXPAs* were up-regulated by about 2.4-fold in response to salt stress. Moreover, “inositol metabolism” pathways related to cell walls were also found to be active. All changes in those genes would cause cell volume to increase. In fact, some very interesting changes that might be related to cellular deformation or enlargement were observed. Specifically, an *Actin* and a gene encoding Annexin (ANXA) were dramatically up-regulated by more than 10-fold. ANXA forms an evolutionarily conserved family of proteins known to be involved in mediating plant stress responses and has been shown to regulate fiber elongation through its interaction with actin^50^. Moreover, some genes in the auxin (AUX) signaling pathway known to be responsible for cell enlargement were also up-regulated. Based on these findings, a new hypothesis that cell enlarging activities benefit salt tolerance in *N. tangutorum* is proposed. It is further speculated that the series of genes described above might co-function through cell enlargement to provide space for the dilution of salt ions in cells, as well as operate in conjunction with osmotic regulators to provide sufficient osmotic pressure for water to enter cells. As a result of these two effects, the intracellular salt ion concentration decreased. Information about DEGs involved in ion homeostasis regulation is given in Supplementary Table S18.

### Signal transduction

Signal transduction plays a pivotal role in enhancing tolerance to environmental stresses. KEGG enrichment analysis showed that genes in the “MAPK signaling pathway—plant” were significantly enriched. Specifically, 12 DEGs in this pathway were found to be up-regulated, including a gene associated with *nucleoside diphosphate kinase* (*NDPK*, 6.6-fold), three *calmodulin* genes (*CALM*, about 7-fold), and seven *protein phosphatase 2C* genes (*PP2C*, about 1.6-fold). However, a gene encoding respiratory burst oxidase (RBOH) in this pathway was down-regulated. The enzyme is also called NADPH oxidase, which is known to oxidize NADPH to produce ROS^51^. The production of ROS is among the early events following stress response in plants, which play a key signal transduction role in cells^51,52^. In our experiment, samples were collected when plants had been treated for 7 days (it was obviously not an early stage of stress response). Therefore, down-regulation of *RBOH* might be involved in ROS balance mechanism in plants^51^. In addition, Ethylene-responsive transcription factors (ERFs) are reportedly involved in transcriptional regulation of multiple signal transduction or stress resistance activities^20^. In our study, four *ERF* genes with different expression patterns were identified. Specifically, two genes were up-regulated and two were down-regulated. These genes might have been involved in the “MAPK signaling pathway—plant” and “Plant hormone signal transduction” pathway.

Phytohormones signaling pathways form an important part of the signaling network involved in responses to diverse stresses^20^. Based on enrichment analysis, the “plant hormone signaling” pathway is significant enriched, and a series of DEGs were involved in the AUX/IAA, ethylene, and jasmonate induced processes. Among these, *ERFs* also belonging to the “MAPK signaling pathway—plant” showed expression differentiation. In addition, as mentioned above in the ion homeostasis section, the AUX pathway was very active, and the *AUX/IAA* and the *small auxin up-regulated RNA* (*SAUR*) genes were up-regulated. However, the *ARF* in the AUX signaling pathway was down-regulated by 4.9-fold. This finding indicates that the *ARF* activities were obviously out of sync with the activities of other genes in the pathway. In fact, *ARF* is a type of TF that regulates the expression of auxin-responsive genes, and the members of *ARF* family could be induced or inhibited by salt stress depending on their function in stress response^53^. Therefore, the *ARF* isolated in this transcriptome might be related in other function rather than phytohormones signaling pathway.

Calcium, which is a second messenger in plants, is widely involved in signal transduction in plant responses to various biological and abiotic stresses^54^. In the cell, calcium signal perception is mainly realized as a result of spatiotemporal variations in its concentration in the cytoplasm^55^. Accordingly, it is crucial to control the intracellular release of Ca^2+^ or entry of Ca^2+^ through the plasma membrane^50^. The ANXAs mentioned above are also Ca^2+^ sensitive proteins, which specifically interact with lipids on the plasma membrane to control entry of calcium^50^. ANXA activities related to Ca^2+^ signal transduction occur upstream in the total cascade reactions^56^; therefore, its expression level is probably not very high when it participates in Ca^2+^ signal transduction. In this study, three *ANXA*s were found to be up-regulated, but to different degrees. Specifically, one was up-regulated by 13-fold, while the other two merely increased by about 1-fold. When combined with the above discussion on the function of *ANXAs*, these findings suggest that the highly up-regulated *ANXA* was mainly responsible for cell elongation activity, while the other two *ANXA*s might be responsible for Ca^2+^ signaling. In addition, many genes encoding proteins containing EF hand were identified and shown to be up-regulated by 3- to 9-fold. Many of these genes likely play important roles in Ca^2+^ signal transduction^56^. Overall, these results indicate that multiple signaling pathways, including the “MAPK signaling pathway—plant,” the “plant hormone signaling” pathway, and the Ca^2+^ signaling pathway co-regulate various physiological and biochemical activities of salt tolerance in *N. tangutoru*^57^. Information about the DEGs involved in signal transduction is displayed in Table 2.

**Table 2:** Information of differentially expressed genes involved in signal transduction.

Abbreviation	Full name	Function in signal transduction	Fold change	Gene number	Sequencing ID	Reference
*PP2C*	*Protein phosphatase 2C*	Inhibited by abscisic acid in one of MPAK signal pathways	1.0–3.2	7	c27292_g1_i1;c34913_g1_i2;c34913_g1_i1;c34913_g1_i4;c34913_g1_i5;c36754_g1_i1;c36754_g1_i2	^58^
*CALM*	*Calmodulin*	Binds to Ca^2+^ and converts to mitogen-activated protein kinase 8 in one of MAPK signal pathways	6.3–7.4	3	c6792_g1_i1;c78451_g1_i1;c4299_g1_i1	^59^
*NDPK2/ndk*	*Nucleoside diphosphate kinase*	Provides GTP for H_2_O_2_-mediated MAPK signal pathway	6.6	1	c71910_g1_i1	^60^
***RBOH***	***Respiratory burst oxidase***	Be inhibited in a MAPK signal pathway; produces ROS in some ROS signal pathways	**− 1.8**	1	c36290_g1_i1	^52,61^
*ERF1/2*	*Ethylene-responsive transcription factor 1/2*	Be split by the ubiquitin mediated proteolysis, and regulates downstream transcriptional activities in an ethylene induced signal pathway or MAPK signal pathways	1.4–1.7	2	c15192_g1_i1;c28875_g1_i2	^62^
***ERF1/2***			**− 2.6 to − 1.9**	2	c15559_g1_i1;c35088_g1_i1	^62^
*IPPK*	*Inositol-pentakisphosphate 2-kinase*	Phosphorylates insP5 to form insP6 in the phosphatidylinositol signal system	1.3	1	c39873_g1_i3	^63^
*ANXA*	*Annexin*	Acts as Ca^2+^ permeable channel in calcium signal pathways	1.3–1.7	2	c30549_g1_i1;c37355_g1_i1	^50,54^
*AUX/IAA*	*Auxin-responsive protein*	Acts as repressors of auxin-induced gene expression in a plant hormone signal pathway	1	1	c34563_g1_i1	^64^
*SAUR*	*Small auxin up-regulated RNA*	Acts downstream of the activity of DNA-binding auxin response factors in a plant hormone signal pathway	1	1	c28798_g1_i1	^64^
***ARF***	***Auxin response factor***	Be split by the ubiquitin mediated proteolysis and regulates downstream transcriptional activities in an auxin induced plant hormone signal pathway	**− 5.1 to − 4.8**	2	c20159_g1_i2;c21885_g1_i1	^64^
***ARR-A***	***Two-component response regulator***	Acts downstream of the activity of DNA binding to two-component response regulator *ARR-B* in one of cytokinin induced plant hormone signal pathway	**− 1.2 to − 1.1**	2	c17257_g1_i1;c28515_g1_i1	^64,65^
***JAZ***	***Jasmonate ZIM domain-containing protein***	Be degraded by SCFCOI1/26S pathway and produces MYC to regulate downstream activities in a jasmonic acid induced plant hormone signal pathway	**− 1.4**	1	c27056_g1_i1	^66^

Negative values are in bold.

### Responses of transcription factors

Transcription factors have been shown to regulate the expression of downstream genes, as well as to impact different physiological characteristics of plants in response to environmental stress^19^. An increasing number of TFs involved in plant responses to abiotic stresses has been discovered in many plant species^62,67–73^. In this study, 24 TFs distributed in eight families were detected as DEGs. Among these, all members of the *Nam-ataf1,2-cuc2* (*NAC*) and *FAR1* families were up-regulated and all *DNA binding with one finger* (*Dof*) family members were down-regulated. Both up and down-regulation were observed in *ERF* family members. Although all members of the *NAC* and *FAR1* families were up-regulated, the degree to which they changed differed. Taken together, these findings indicate the three TF families (*ERF*, *FAR1*, and *NAC*) might play multiple functional roles in the salt tolerance of *N. tangutorum*. Information about the DEGs involved in TFs is displayed in Supplementary Table S19.

## Conclusion

Under the present investigated conditions and analytical method, 1419 DEGs were identified. Annotation and enrichment analysis revealed these DEGs were mainly involved in various stress response-related processes, including energy metabolism, antioxidant activities, signal transductions, transcriptional regulations and maintenance of ion homeostasis. It's worth noting that flavonoids, especially anthocyanins, might be important antioxidants. Additionally, a series of activities such as osmotic regulation, increasing the thickness of the cuticle, cell expansion were involved in keeping ion homeostasis. Finally, the high light of the paper is numerous identified genes were found to be participated in cell elongation, though separate physiological activities related to cell enlargement under salt stress have been reported, but such comprehensive and synchronous evidence (cytoskeleton remodeling activities, cell wall reconstruction activities, and auxin activities) has never been reported. In conclusion, the results presented here will be useful for understanding salt tolerance mechanisms in *N. tangutorum* and expanding knowledge regarding the salt tolerance mechanisms of other halophytes, thereby facilitating their effective development and utilization.

## Supplementary Information

Supplementary Figures.

Supplementary Information 1.

Supplementary Information 2.

## Data Availability

The datasets generated during and/or analyzed during the current study are available from the corresponding author J. W. on reasonable request.
