# Supplementary material for: Transcriptome analysis of halophyte Nitraria tangutorum reveals multiple mechanisms to enhance salt resistance
Source: Sci Rep. 2022 Aug 18;12:14031. doi: 10.1038/s41598-022-17839-z (PMC9388663; doi:10.1038/s41598-022-17839-z)
Supplement: Supplementary file 1 — Supplementary Figures. [file 41598_2022_17839_MOESM1_ESM.pdf]

CK-1_1.clean.fq.gz:

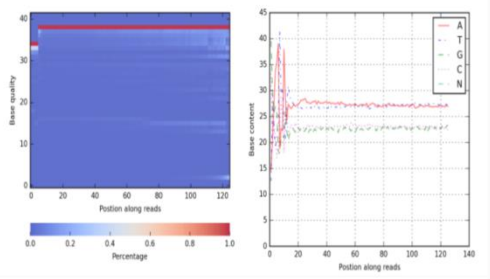

CK-1_2.clean.fq.gz:

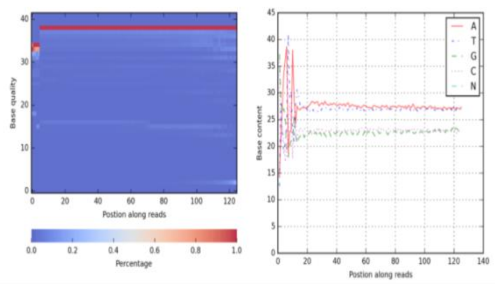

CK-2_1.clean.fq.gz:

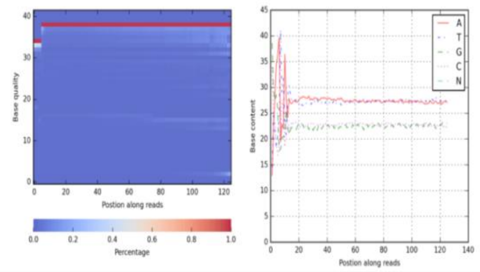

CK-2_2.clean.fq.gz:

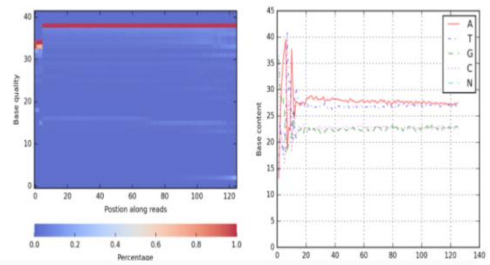

CK-3_1.clean.fq.gz:

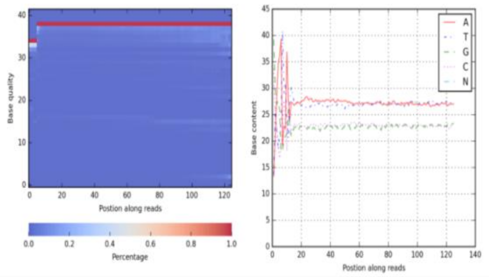

CK-3_2.clean.fq.gz:

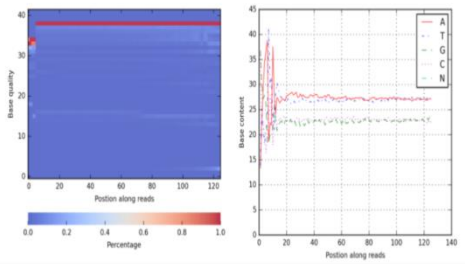

T-1_1.clean.fq.gz:

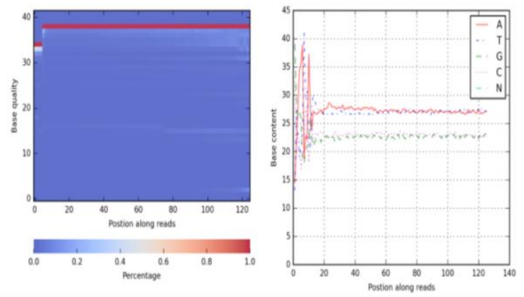

T-1_2.clean.fq.gz:

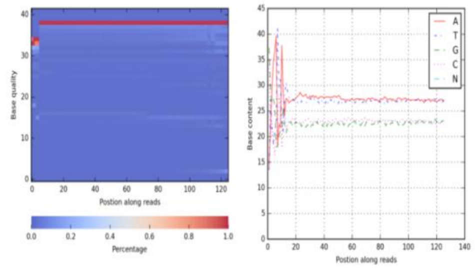

T-2_1.clean.fq.gz:

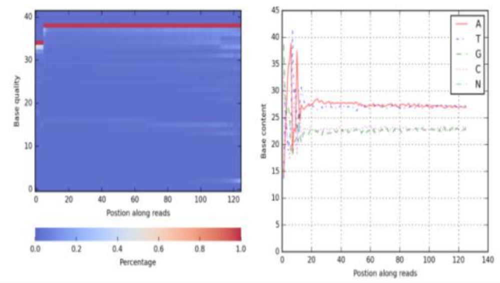

T-2_2.clean.fq.gz:

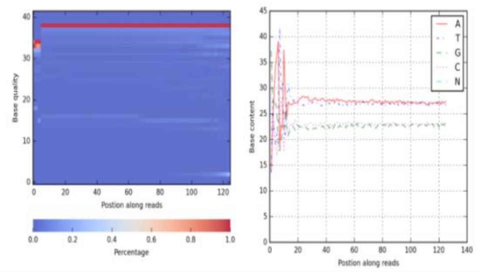

T-3_1.clean.fq.gz:

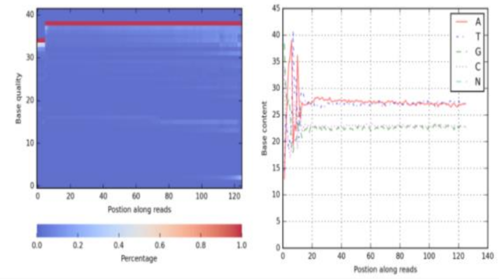

CK-1_2.clean.fq.gz:

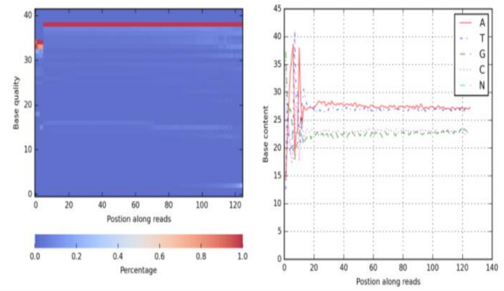

Figure S1 Sample sequencing quality and base distribution

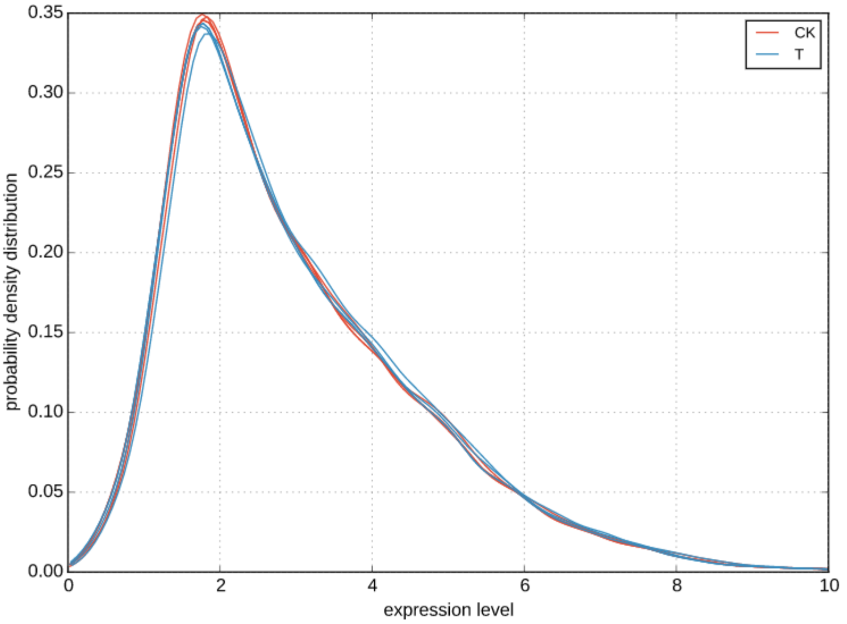

Figure S2 Sample expression level probability density distribution.

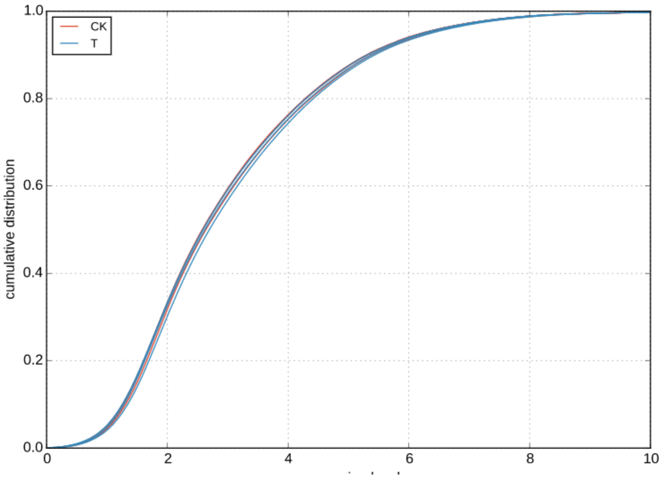

Figure S3. Cumulative probability density distribution of sample expression level.

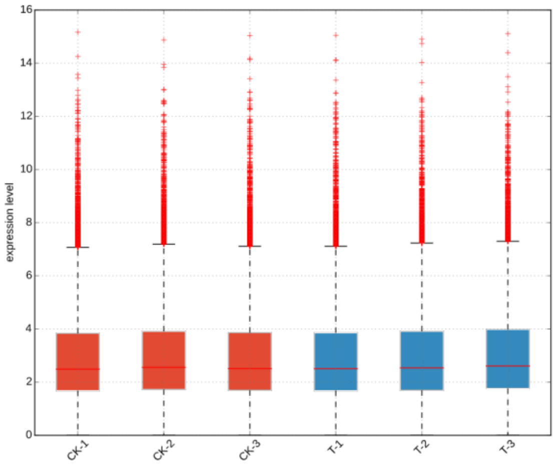

Figure S4. Horizontal box diagram of sample expression.

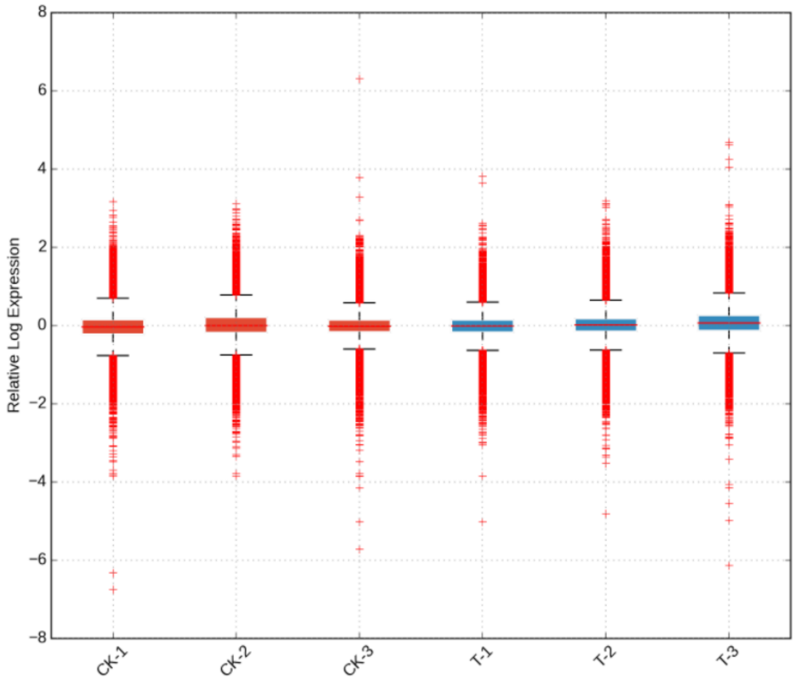

Figure S5. Comparison of relative expression levels of samples.

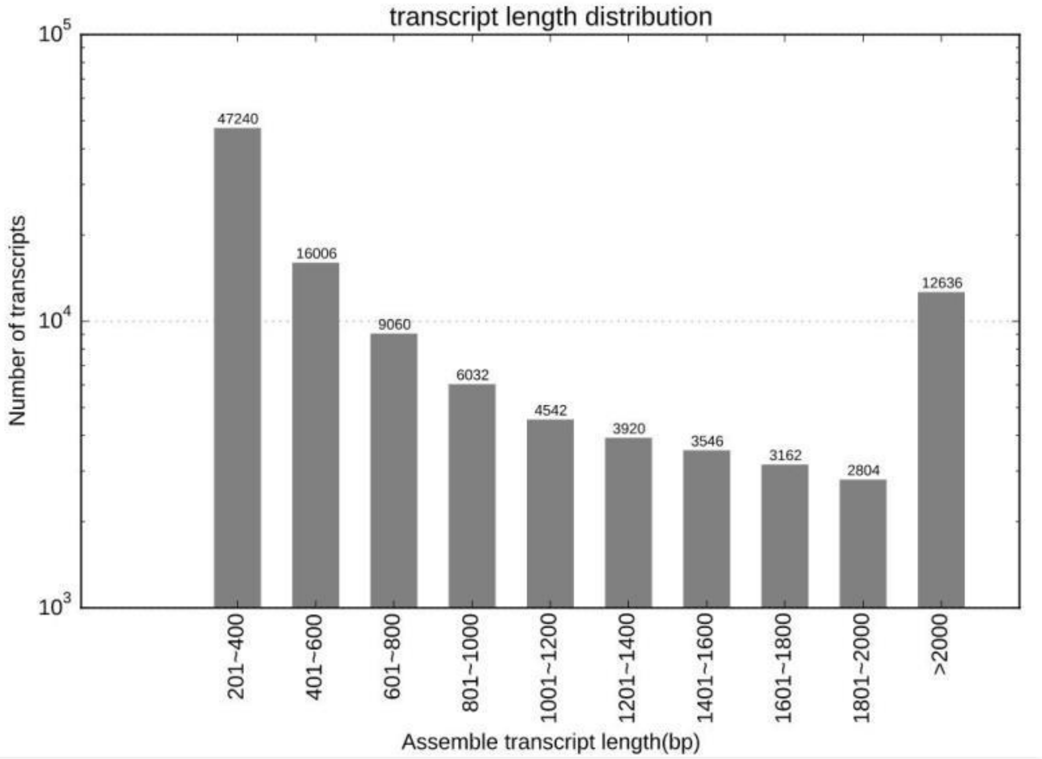

Figure S6. Length distributions of transcripts

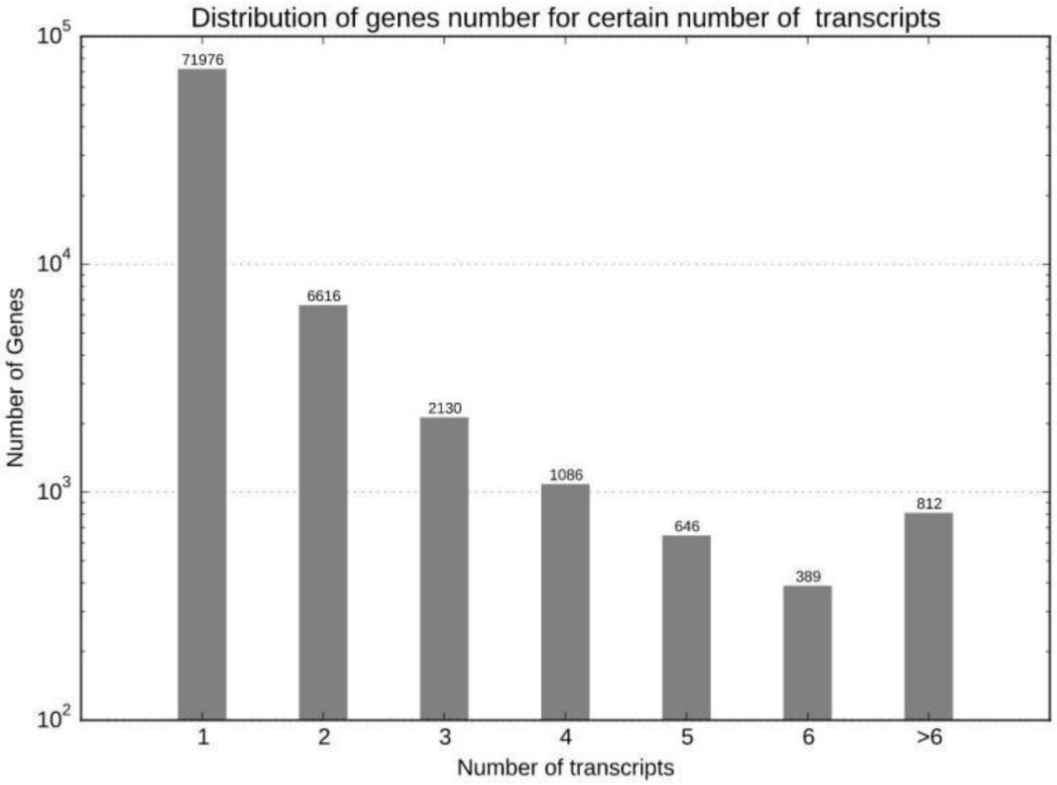

Figure S7. Distribution of unigene numbers for certain numbers of transcripts

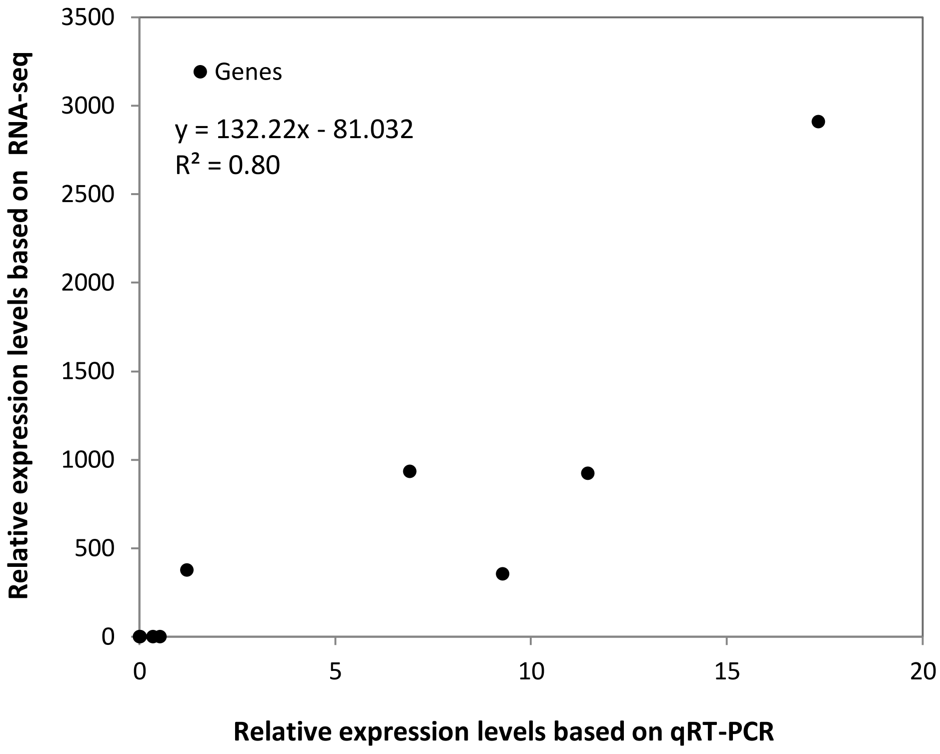

Figure S8. Scatter plot of correlation between Relative expression levels based on RNA-seq and qRT-PCR. Relative expression levels based on RNA-seq were represented by FC values, and relative expression levels based on qRT-PCR are represented by $2^{-\Delta\Delta Ct}$ values.

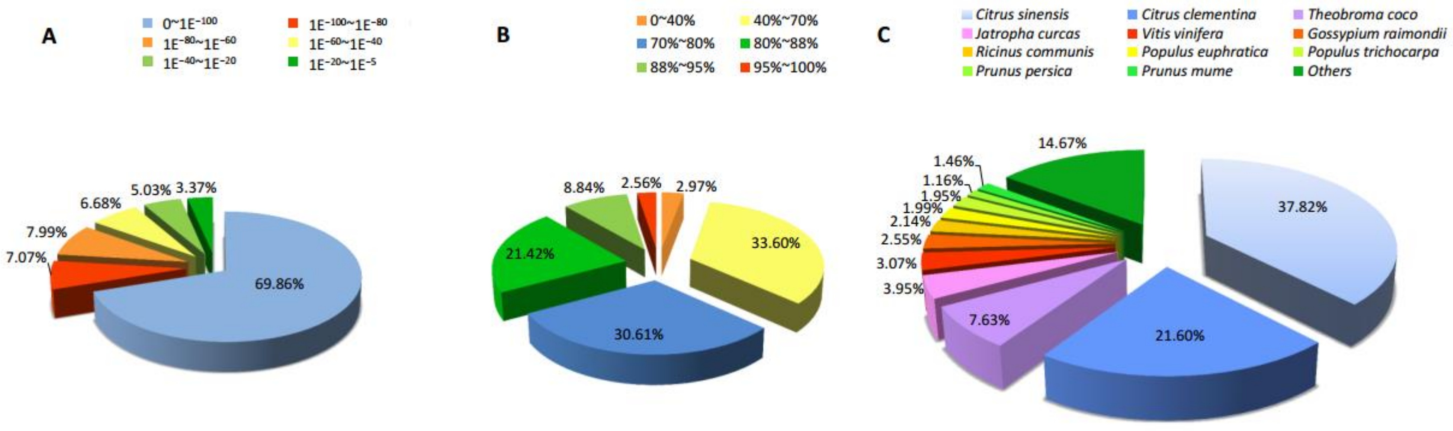

Figure S9. (a) Similarity search of unigenes against Nr databases. (b) E-value distribution of Nr annotation results. (c) Similarity distribution of Nr annotation results. Species distribution of Nr annotation results.

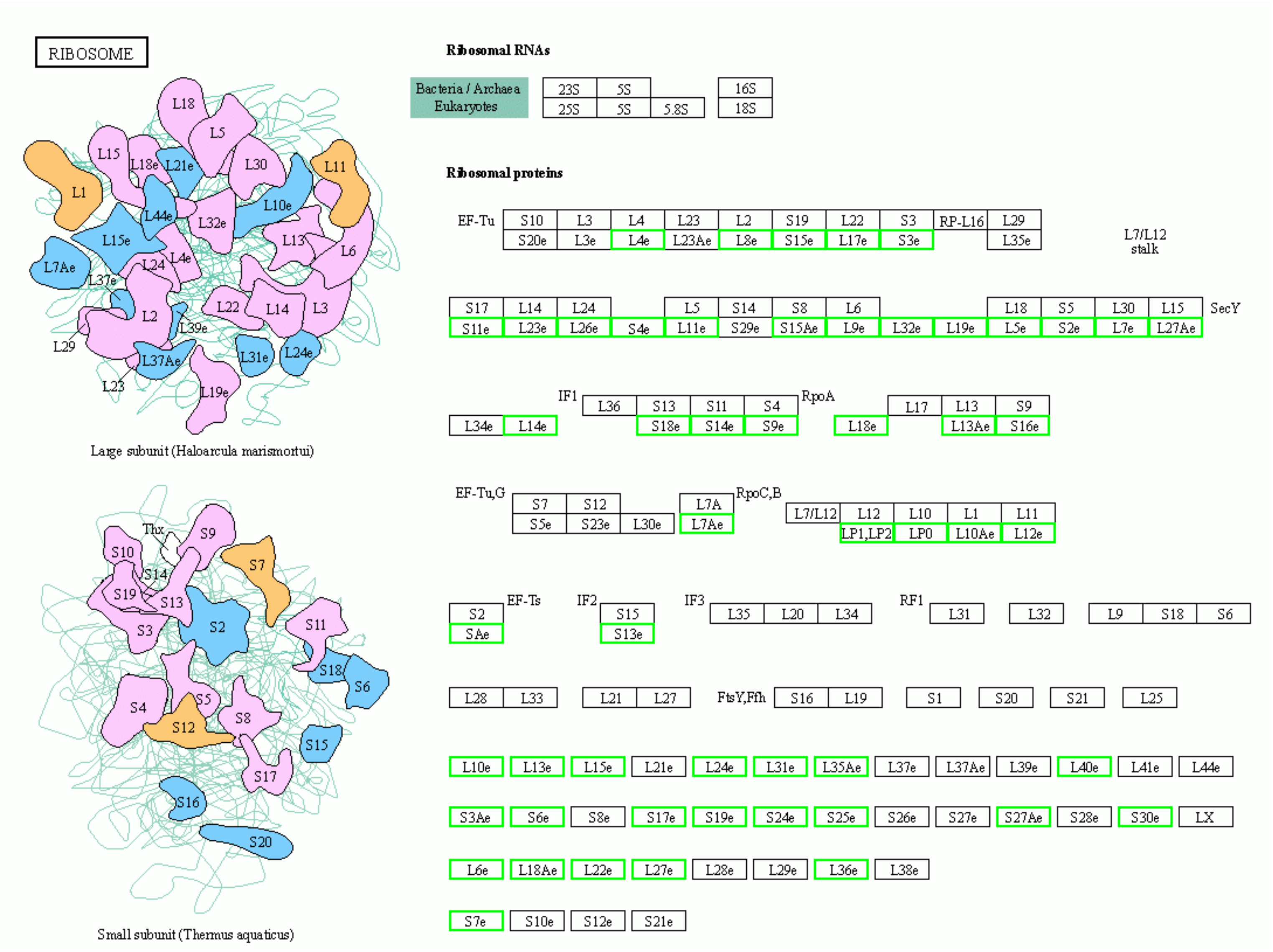

Figure S10. Overview of salinity stress-responsive genes involved in ribosomal biosynthesis in *N. tangutorum* plants.
